# Characterisation of receptor binding by the chemotaxis inhibitory protein of *Staphylococcus aureus* and the effects of the host immune response

**DOI:** 10.1016/j.molimm.2006.12.022

**Published:** 2007-04

**Authors:** Andrew J. Wright, Adrian Higginbottom, Didier Philippe, Abhishek Upadhyay, Stefan Bagby, Robert C. Read, Peter N. Monk, Lynda J. Partridge

**Affiliations:** a Department of Molecular Biology and Biotechnology, University of Sheffield, Sheffield S10 2TN, UK; b School of Medicine and Biomedical Science, University of Sheffield, Sheffield S10 2RX, UK; c Department of Biology and Biochemistry, University of Bath, Bath BA2 7AY, UK

**Keywords:** Complement, Receptor, *Staphylococcus aureus*, Antibody, C5a

## Abstract

The chemotaxis inhibitory protein of *Staphylococcus aureus* (CHIPS) is reported to bind to the receptors for C5a and formylated peptides and has been proposed as a promising lead for the development of new anti-inflammatory compounds. Here we have examined the receptor specificity and mode of action of recombinant CHIPS_28–149_ and also the immune response to CHIPS_28–149_ in patients with *S. aureus* infections and in uninfected controls. Recombinant CHIPS_28–149_ bound with high affinity to the human C5a receptor (C5aR), but had low affinity for the second C5a receptor, C5L2, and the formyl peptide receptor, FPR. Although ligand binding to C5aR was potently inhibited, CHIPS_28–149_ had much weaker effects on ligand binding to C5L2 and FPR. Similarly, CHIPS_28–149_ potently inhibited the ligand-induced activation of C5aR but was less potent at inhibition via FPR. NMR studies showed that CHIPS_28–149_ bound directly to the N-terminus of C5aR but not C5L2, and CHIPS_28–149_ residues involved in the interaction were identified by chemical shift analysis. All human sera examined contained high titres of IgG and IgA reactivity against CHIPS_28–149_, and no correlation was observed between infection status at the time of serum collection and antibody titre. Individual serum samples promoted or inhibited the binding of CHIPS_28–149_ to C5aR, or had no effect. IgG depletion of serum samples abrogated the effects on CHIPS binding, demonstrating that these were antibody mediated. Sera from infected individuals were more likely to inhibit CHIPS_28–149_ binding than sera from healthy controls. However, high antibody titres correlated well with both inhibition and enhancement of CHIPS_28–149_ binding to C5aR; this suggests that the inhibitory effect relates to epitope specificity rather than greater antibody binding. We conclude that CHIPS is likely to be too immunogenic to be used as an anti-inflammatory treatment but that some antibodies against CHIPS may be useful in the treatment of *S. aureus* infections.

## 1 Introduction

C5a is the most potent pro-inflammatory mediator produced in the complement cascade (Guo and Ward, 2005), and is a potent chemoattractant for all myeloid cells (Kohl, 2001). Binding of C5a to its receptor induces a range of inflammatory effects including leukocyte recruitment and chemotaxis, upregulation of leukocyte adhesion molecule expression (CD18, ICAM1), release of proteolytic and reactive oxygen and nitrogen species, cytokine production, activation of the coagulation cascade, contraction of smooth muscle and changes in vascular diameter and permeability (reviewed in Gerard and Gerard, 1994; Guo and Ward, 2005). The receptor for C5a, C5aR, belongs to the rhodopsin family of seven transmembrane G-protein coupled receptors (Boulay et al., 1991; Gerard and Gerard, 1991). The binding of C5a to the C5aR is postulated to occur via a ‘two-site binding’ mechanism (DeMartino et al., 1994): the basic core of C5a is thought to interact with acidic residues in the receptor N-terminus (Mery and Boulay, 1994), while the C-terminal domain of C5a binds in a pocket formed by largely hydrophobic residues within the transmembrane helices of the C5aR (Higginbottom et al., 2005). Recently C5a-like receptor 2 (C5L2), which shares 35% amino acid identity with C5aR, was shown to bind both C5a and C5a-des-Arg, although with a 10-fold higher affinity for the stable metabolite, C5a des-Arg (Cain and Monk, 2002). *S. aureus* supernate (SaS) contains components that cause a decreased chemotactic activity of neutrophils toward C5a and/or N-formyl peptides (Veldkamp et al., 2000). The factor responsible for this activity, ‘Chemotaxis Inhibitory Protein of *Staphylococcus aureus*’ (CHIPS), is a 14.1 kDa protein (Postma et al., 2004) found in over 60% of *S. aureus* clinical isolates and is located on the bacteriophage encoded pathogenicity island SaPI5. It has been suggested that CHIPS could be exploited as an anti-inflammatory therapeutic agent (de Haas et al., 2004). Residues Asp10, Gly12, Asp15, and Asp18 in the N-terminal domain of C5aR are crucial for the interaction with CHIPS (Postma et al., 2005). A CHIPS_31–121_ fragment showed the same C5aR blocking activity as intact CHIPS although this fragment did not block FPR binding, suggesting that the FPR binding site is at the extreme N-terminus of CHIPS (Haas et al., 2004).

We have produced recombinant CHIPS_28–149_ to characterise the mechanism of action of CHIPS and to assess the antibody responses of controls and *S. aureus*-infected patients. CHIPS_28–149_ was found to be a potent competitive antagonist at C5aR with rapid binding kinetics but was only weakly active at C5L2 or FPR. All sera tested contained high anti-CHIPS_28–149_ antibody titres and approximately half of these affected the binding to C5aR. Anti-CHIPS_28–149_ antibodies that block CHIPS binding may have therapeutic potential in the treatment of *S. aureus* infections.

## 2 Methods and materials

### 2.1 Proteins and peptides

DNA coding for CHIPS residues 28–149 (CHIPS_28–149_) was amplified from N315 MRSA strain genomic DNA and cloned into a modified pGEX4T1 vector (Sheffield et al., 1999) using 5′-CAT GCC ATG GCT TTT ACT TTT GAA CCG TTT-3′ and 5′-CCG CTC GAG CTA TTA GTA TGC GTA TTC ATT AGT TT-3′ primers. GST-CHIPS_28–149_ was overexpressed using BL21 (DE3) cells with IPTG induction. Cells were lysed by sonication and GST-CHIPS_28–149_ was batch purified on glutathione sepharose 4B resin according to manufacturer's instructions (GE Healthcare). After removal of the GST carrier protein using TEV protease, CHIPS was further purified on a Mono S cation exchange column (GE Healthcare) using an AktaPurifier 10 chromatography unit (GE Healthcare), and was at least 95% pure as estimated by SDS PAGE. ^15^N- and ^13^C, ^15^N-labelled samples of CHIPS_28–149_ for NMR spectroscopy were produced by growing cells on M9 medium supplemented with 1 g l^−1^ ^15^N-NH_4_Cl and 1 g l^−1^ ^15^N-NH_4_Cl/2 g l^−1^ U-^13^C_6_-glucose as the sole nitrogen and carbon sources. Protein expression in minimal medium was induced using 0.5 mM IPTG and cells were harvested after overnight induction at 37 °C. Isotope incorporation was about 96% for both ^15^N and ^13^C, as judged by mass spectrometry. Recombinant human C5a protein (rh-C5a) was expressed and purified according to a previously described protocol (Paczkowski et al., 1999). fMLP was bought from Sigma–Aldrich. Human C5aR peptides corresponding to the N-terminal extracellular region M1-D37 with an additional -APAPAC on the C-terminus (used for generating immune serum) and extracellular region R174-R206 with the same additional sequence at the C-terminus (this had C188 changed to a Ser to prevent disulphide bond formation with the C-terminal Cys) were a generous gift from Dr M. Barker, Division of Genomic Medicine, Sheffield, UK. Protein concentrations were determined by measuring absorbance at 278 nm in denaturing conditions and using standard values of extinction coefficients for Trp, Tyr and Phe residues (Edelhoch et al., 1967).

### 2.2 NMR assignment of CHIPS_28–149_

NMR spectra of CHIPS_28–149_ were recorded at 25 °C on a Varian Unity Inova 600 MHz spectrometer. Backbone assignment was carried out using ^1^H–^15^N HSQC (Kay et al., 1992), HNCA, HN(CO)CA, HNCACB, CBCA(CO)NH, HNCO, and HNHA data sets (Cavanagh et al., 1996; Grzesiek, 1992; Kay et al., 1992; Kuboniwa et al., 1994; Muhandiram, 1994; Wishart et al., 1995). Chemical shifts were referenced to DSS (Wishart et al., 1995). NMR data were processed using nmrPipe (Delaglio et al., 1995) and Sparky (Goddard and Kneller, 2001) was used for NMR data analysis. NMR samples were made up in 100 mM phosphate pH 7.0, 200 mM NaCl, 1 mM DTT and 0.5 mM EDTA.

### 2.3 Analysis of CHIPS_28–149_ interactions: NMR chemical shift perturbation

To help determine the mechanism by which CHIPS functions, several potential binding partners were titrated into separate NMR samples of uniformly ^15^N-labelled CHIPS_28–149_: the chemoattractants human C5a and formylated peptide fMLP, peptides corresponding to two extracellular regions of the human C5a receptor (the N-terminal 37 residues, hC5aR_1–37_, and residues 174–206, hC5aR_174–206_, corresponding to the second extracellular loop of the receptor) and a peptide corresponding to residues 1–32 of human C5L2, a C5a receptor homologue, were titrated in turn with ^15^N-labelled CHIPS_28–149_. A ^1^H–^15^N HQSC spectrum of ^15^N-labelled CHIPS_28–149_ was recorded after each addition of unlabelled lyophilized ligand to the particular CHIPS_28–149_ sample. The CHIPS_28–149_ concentration for these experiments was 0.7 mM. The titration points corresponded to ligand:CHIPS_28–149_ molar ratios of 0, 1:2, 3:4, 1:1 and 5:4. Since the ligands were added as lyophilized powder, the total volume of the sample, and thus CHIPS concentration in the sample, was assumed to change negligibly throughout the experiments. The sample pH was monitored throughout the titrations. In order to quantify the CHIPS_28–149_-hC5aR_1–37_ interaction, the peak position and linewidth at each point in the titration were estimated by fitting Lorentzian functions to each individual resonance, using the program Sparky (Goddard and Kneller, 2001). Resonances that experienced chemical shift perturbation during the titration, but little or no change in linewidth, were analysed in terms of a simple bimolecular association. For one-to-one binding of a protein (*P*) to a ligand (*L*), when the fast exchange condition is satisfied, the observed chemical shift difference Δ*δ*_obs_ as described by Lian and Roberts (1993), where *P*_T_ and *L*_T_ are the total concentrations of protein and ligand, respectively, (*δb* − *δf*) is the total chemical shift difference between the bound and free state, and *K*_d_ is the equilibrium dissociation constant. *P*_T_, *L*_T_ and (*δb* − *δf*) are all measurable, hence *K*_d_ can be determined. Residues for which the chemical shift change evolution did not match an exponential curve were discarded from the calculations. The curve fitting was done manually for each shifted residue by iterative assignment using Microsoft Excel.

### 2.4 Analysis of CHIPS interactions: isothermal titration calorimetry (ITC)

ITC experiments were carried out by Margaret Nutley at the BBSRC/EPSRC Biological Microcalorimetry Facility, Department of Chemistry, University of Glasgow. CHIPS_28–149_ (0.2 mM) was titrated with the peptides hC5aR_1–37_ and hC5aR_174–206_.

### 2.5 Transfection and cell culture

RBL-2H3 and CHO cells were routinely cultured in Dulbecco's modified Eagle's medium + 10% (v/v) fetal calf serum (supplemented with 400 mg/L G-418 for transfected cells) at 37 °C, 8% CO_2_. The cDNA for ChemR23, FPRL1 and FPRL2 was purchased from UMR cDNA Resource Center (www.cdna.org) in pcDNA3.1. Cells were transfected with chemoattractant receptors by electroporation, as previously described (Crass et al., 1999).

### 2.6 Flow cytometric receptor binding studies

For ligand binding studies, CHO cells transfected with the appropriate receptor (50,000 per well of a 96-well microtitre plate) were incubated with the stated concentrations of His_6_-tagged C5a or C5a des Arg for 30 min at 4 °C, in PBS + 0.1% BSA, 0.2% (w/v) NaN_3_, then washed twice with cold PBS to remove unbound ligand (Wilken et al., 1999). The cells were incubated with anti-RGSHis_6_ antibody for 30 min at 4 °C, washed with PBS and anti-mouse IgG-FITC (Sigma) used to detect bound ligand by FACS analysis (FACSort, Becton Dickinson). The association of CHIPS with C5aR was analysed by adding FITC-CHIPS_28–149_ to C5aR-transfected RBL cells (final concentration of 300 nM) followed by an excess of unlabelled CHIPS_28–149_ (14 μM). The dissociation rate, *K*_d_, was calculated using GraphPad Prism v4.0, using the observed association rate (*k*_obs_) and the dissociation rate (*k*_off_) to firstly calculate *k*_on_: *k*_on_ = *k*_obs_ − *k*_off_/[labelled ligand], and secondly: *K*_d_ = *k*_on_/*k*_off_.

### 2.7 Effect of human antibodies on CHIPS binding

CHO or RBL2H3 cells transfected with human C5aR were used for these studies. Dilutions of human serum in PBS + 0.1% BSA, 0.2% (w/v) NaN_3_ were pre-incubated for 45 min with a fixed concentration of FITC-CHIPS_28–149_ (210 nm, determined to give 50% maximal binding). Binding of the FITC-CHIPS_28–149_ to the transfected cells was then determined by flow cytometry as described above. In some instances, human IgG purified using proteinG-Sepaharose (Amersham, UK), concentrated to ∼12 mg/ml using Vivaspin 20 columns (Pierce, UK) and deaggregated by centrifuging at 100,000 × *g* for 1 h, was used. In other cases, serum samples were depleted of IgG by incubating them with an equal volume of protein G-Sepharose on a rotary mixer overnight at 4 °C prior to use in the assay.

### 2.8 Inhibition of receptor activation by rCHIPS_28–149_

Receptor activation in RBL cells was measured as the release of β-hexosaminidase from intracellular granules, as described (Cain et al., 2000). The percentage of β-hexosaminidase release was calculated as a percentage of the release in the absence of CHIPS_28–149_. Total β-hexosaminidase content was determined following cell lysis with 0.1% NP-40. Assay of the antagonist activity was performed as described above except that the antagonists were added at varying concentrations for 15 min before the addition of C5a or C5a des-Arg_74_ at a final concentration of 50 or 250 nM, respectively. IC_50_, EC_50_ and standard error values were obtained by non-linear regression analysis using GraphPad Prism 4.0.

### 2.9 ELISA titration of human serum

Serum samples were obtained from infected patients or ‘normal’ volunteer donors recruited under the approval of the South Sheffield Research Ethics Committee (Study No. 02/299). Samples from infected patients were taken after recent (less than 4 weeks) acute *S. aureus* infection, as confirmed by bacteriological culture of the infectious agent. CHIPS_28–149_ was coated to 96 well microtitre-plates (Nunc MaxiSorp, Denmark) at 10 μg ml^−1^ in carbonate/bicarbonate buffer (pH 9.6) overnight at 4 °C. Following blocking in 0.2% gelatin/PBS, plates were used to titrate human serum using anti-human IgG, IgM, or IgA alkaline-phosphatase labelled secondary antibodies (Sigma). Plates were developed using pNPP (Sigma, UK) substrate and OD_405 nm_ recorded by spectrophotometry.

## 3 Results

### 3.1 Production and characterisation of recombinant CHIPS

**Fig. 1:**
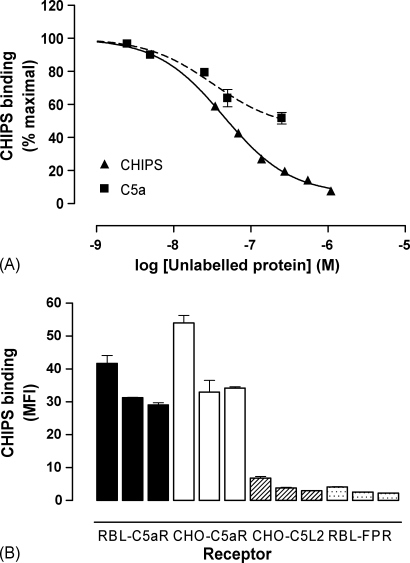
Recombinant CHIPS binds specifically to the human C5a receptor. (A) Cells transfected with human C5a receptor were pre-treated with the stated concentrations of unlabelled CHIPS or human C5a before incubation with 210 nM FITC-rCHIPS_28–149_. (B) Binding of 6, 1.2, and 0.6 μM FITC-labelled rCHIPS_28–149_ to chinese hamster ovary (CHO) or rat basophilic leukemia (RBL) cells transfected with C5a receptor (C5aR), C5L2 or formyl peptide receptor (FPR). Cell-associated fluorescence was measured by flow cytometry. Data shown are means of three separate experiments performed in duplicate, ±S.E.M.

**Fig. 2:**
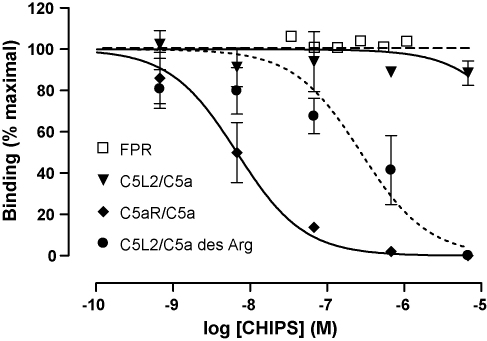
Inhibition of ligand binding by CHIPS. Cells transfected with C5a receptor (C5aR), C5L2 or formyl peptide receptor (FPR) were co-incubated with the stated concentrations of rCHIPS_28–149_ with His_6_-C5a (50 nM), His_6_-C5a des Arg (50 nM) or FITC-formyl peptide (500 nM). Data shown are the means of three separate experiments performed in duplicate, ±S.E.M.

**Fig. 3:**
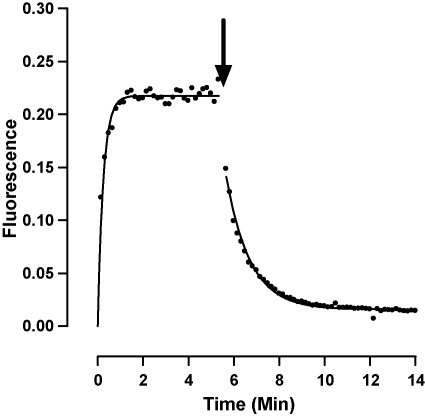
Kinetic analysis of ligand binding to chemoattractant receptors. The kinetics of FITC-rCHIPS_28–149_ binding to cells transfected with C5a receptors was measured using flow cytometry after addition of the fluorescent ligand to RBL cells transfected with C5aR at 300 nM. Dissociation was initiated by the addition of 14 μM unlabelled ligand (arrow): Data are shown as geometric means of fluorescence values obtained in 0.1 s time windows and is one experiment, typical of five performed.

**Fig. 4:**
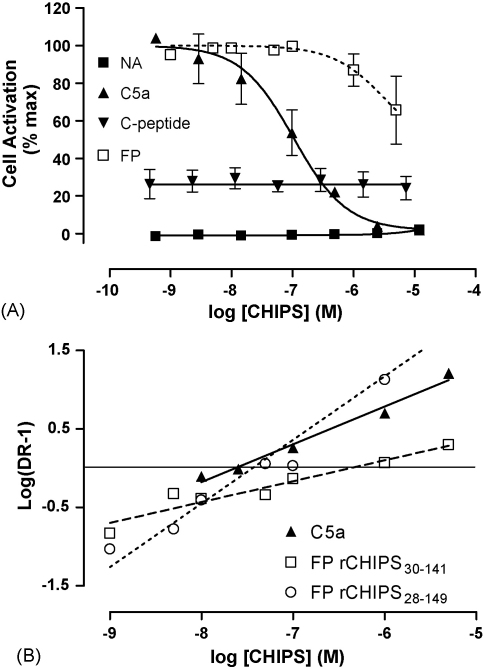
CHIPS inhibits the activation of C5aR and FPR. (A) RBL cells transfected with C5a receptor or formyl peptide receptor were pre-treated with rCHIPS_28–149_ at the stated concentrations before the addition of 10 nM formyl peptide (FP), 10 nM C5a, 100 μM C5a C-terminal peptide (C-peptide) or vehicle alone (NA). Cell activation was assessed as the secretion of β-hexosaminidase, and is expressed as a percentage of the maximal response to C5a (C5a, C-terminal peptide, NA) or formyl peptide (FP). Data are means of two to three experiments performed in duplicate ± S.D. (B) Data from inhibition experiments was used to draw Schild plots to analyse the effects of rCHIPS_28–149_ on C5a and formyl peptide receptors. In a further set of three experiments, rCHIPS_30–141_ was used on formyl peptide receptors.

Recombinant (r)CHIPS_28–149_ was produced in *E. coli* as a fusion protein with GST, which was cleaved using TEV protease to leave three N-terminal residues, GlyAlaMet, from the vector. CHIPS is reported to bind to human C5aR and FPR (Haas et al., 2004) and so we determined the functionality of rCHIPS_28–149_ by characterising the binding of an FITC-labelled form to RBL cells transfected with human C5aR (RBL-C5aR). Unlabelled rCHIPS_28–149_ could compete with FITC-rCHIPS_28–149_ for binding to RBL-C5aR cells, with an IC_50_ value of ∼69 nM (Fig. 1A). Similarly, unlabelled rhC5a could also compete with FITC-rCHIPS_28–149_ (Fig. 1A) although even at the high dose of 250 nM, rhC5a failed to compete for more than 50% of the binding sites. FITC-rCHIPS_28–149_ binding was quite specific for C5aR expressed on RBL and CHO cells; binding to the second C5a receptor, C5L2, and the formyl peptide receptor, FPR, was just above the lower limit of detection at 6 μM and undetectable at lower concentrations whereas binding to C5aR was high, even at 0.6 μM (Fig. 1B). No binding to FPRL1, FPRL2 or ChemR23 was detected (data not shown). This result was surprising in light of the similarity of the N-termini of C5aR and C5L2 (Cain and Monk, 2002), and the previously reported binding of CHIPS to FPR (Postma et al., 2004). In further experiments, the ability of CHIPS_28–149_ to prevent ligand binding to C5L2 and FPR was investigated. Although CHIPS_28–149_ could clearly inhibit the binding of C5a to C5aR (Fig. 2A, IC_50_ = 6.7 nM), no inhibition of C5a binding to C5L2 was observed, even at 100 μM CHIPS_28–149_. In contrast, the binding of C5a des Arg to C5L2 could be inhibited (Fig. 2, IC_50_ = 274 nM), suggesting that CHIPS might also be active at C5L2. Using FITC-labelled formyl peptide fMLP, we could not detect any inhibition of binding, even at concentrations of CHIPS_28–149_ that completely inhibit C5a binding to C5aR (Fig. 2). The kinetics of binding to C5aR were also examined, using flow cytometry to measure the association of FITC-CHIPS_28–149_ with RBL-C5aR. Binding reached maximal levels in <2 min (Fig. 3, *t*_1/2_ = 0.17 min), and the addition of an excess of unlabelled CHIPS_28–149_ caused complete dissociation (*t*_1/2_ = 0.73 min). Using data from five separate experiments, *K*_d_ = 7.22 ± 6.66 nM, similar to the figure calculated from competition experiments. Finally, rCHIPS_28–149_ was tested in cell activation studies, using the secretion of β-hexosaminidase to measure the degree of inhibition of receptor activation. CHIPS_28–149_ could inhibit the activation of C5aR by C5a (Fig. 4A, IC_50_ = 2.44 μM), but not by the C-terminal C5a analog EP-54 or by phorbol ester/calcium ionophore, which directly activates cells (Fig. 4A). In contrast, only very weak inhibition of FPR was observed (Fig. 4A, IC_50_ > 1 mM), although at high rCHIPS_28–149_ concentrations, some non-specific inhibition could be observed. We further analysed the mechanism of inhibition of C5aR using Schild plots (Arunlakshana and Schild, 1959) (Fig. 4B). The relationship between the log of the concentration of rCHIPS_28–149_ and the dose-ratio is linear, which suggests that inhibition is reversible and competitive. However, slopes are <1 in all cases, suggesting that negative cooperativity may be occurring. Interestingly, we also tested rCHIPS_30–141_, lacking two N-terminal residues, for the ability to inhibit FPR activation and found that it had 12-fold higher potency at FPR than rCHIPS_28–149_ (Fig. 4B). Taken together, this data suggests that rCHIPS_28–149_ is highly specific for C5aR with low activity at the closely related receptor, C5L2, but no activity at FPR.

### 3.2 Analysis of CHIPS interactions: NMR chemical shift perturbation

**Fig. 5:**
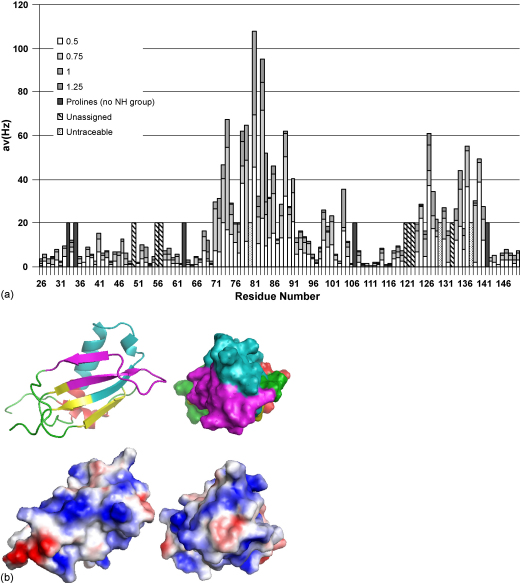
Analysis of the interaction of CHIPS with the N-terminus of C5aR. (a) Averaged chemical shift changes of the amide cross peaks of ^15^N CHIPS_28–149_ upon complex formation with human C5aR_1–37_ peptide, corresponding to the N-terminal extracellular region of the C5a receptor. The chemical shift changes were calculated using the formula $\Delta \delta_{\text{av}}=\sqrt{\left(\Delta \delta^{2}\text{NH}+(\Delta \delta^{2}\text{N}/25)\right)}$, where the shift differences (Δ*δ*) between free and complexed form are observed by two-dimensional ^1^H–^15^N HSQC spectra. (b) Mapping of CHIPS regions interacting with hC5aR_1–37_. Top panels: residues 71–97 (cyan) and 123–140 (magenta), shown by NMR chemical shift perturbation to be involved in interaction with hC5aR_1–37_, are mapped on to the structure of rCHIPS_31–121_ (Haas et al., 2005). The rest of the rCHIPS_31–121_ structure is displayed in yellow (β-strands), red (α-helices), and green (loops). Bottom panels: electrostatic surface view of the representations above. Blue and red represent positively and negatively charged surface.

Using standard triple resonance methodology (Cavanagh et al., 1996; Grzesiek, 1992; Kay et al., 1992; Kuboniwa et al., 1994; Muhandiram, 1994; Wishart et al., 1995), sequence specific backbone resonance assignments (including amide ^1^H and ^15^N, ^13^Cα, ^13^Cβ, ^13^C(O) and ^1^Hα) were made for 95% of non-Pro, non-N-terminal amino acids in CHIPS_28–149_. Titration of ^15^N-labelled CHIPS_28–149_ with either of the chemoattractants C5a and fMLP or the hC5aR_174–206_ peptide caused negligible change in chemical shift or intensity of the peaks in the ^1^H–^15^N HSQC spectrum of CHIPS_28–149_. In contrast, titration of CHIPS_28–149_ with the hC5aR_1–37_ peptide caused significant chemical shift changes in the CHIPS_28–149_^1^H–^15^N HSQC spectrum. These results indicate that CHIPS_28–149_ can bind to the extracellular N-terminal region of the human C5a receptor but does not show significant affinity for C5a, fMLP or the second extracellular loop of hC5aR. The interaction of CHIPS_28–149_ with the N-terminal region (1–32) of the C5aR homologue, C5L2, was also investigated. Interestingly, no chemical shift changes were observed in the CHIPS_28–149_^1^H–^15^N HSQC spectrum even at a 1.5:1 hC5L2_1–32_:CHIPS_28–149_ ratio (data not shown), suggesting that CHIPS_28–149_ does not bind to the N-terminal region of hC5L2. To map the binding site of hC5aR_1–37_ on CHIPS_28–149_, the differences in amide ^1^H and ^15^N chemical shifts between the free and the complexed state were measured. The chemical shift differences were minimized using the formula $\Delta \delta_{\text{av}}=\sqrt{\left(\Delta \delta^{2}\text{NH}+(\Delta \delta^{2}\text{N}/25)\right)}$, where Δ*δ*NH and Δ*δ*N are the difference in the backbone amide ^1^H and ^15^N chemical shifts between the free and complexed state. This approach provides the actual value or an underestimate of the chemical shift perturbation. This analysis indicated that hC5aR_1–37_ binding mainly affected two regions of the CHIPS molecule (Fig. 5a and b): the first group of affected residues, encompassing residues 71–91 (and possibly other amino acids beyond 91), is most strongly affected by the binding. The other region, between residues 123 and 140, shows a smaller but still significant chemical shift perturbation. In order to quantify the CHIPS_28–149_–hC5aR_1–37_ interaction, the peak position and line width at each point in the titration were estimated by fitting Lorentzian functions to each individual resonance. An average *K*_d_ of 8.25 μM was obtained with a standard deviation of 8.60 μM.

### 3.3 Analysis of CHIPS interactions: isothermal titration calorimetry (ITC)

ITC confirmed the binding of hC5aR_1–37_ to rCHIPS_28–149_, while titration of hC5aR_174–206_ with CHIPS_28–149_ did not generate any response from the calorimeter (data not shown). Curves were fitted by non-linear regression analysis: the binding data for hC5aR_1–37_ with CHIPS_28–149_ revealed a *K*_a_ of 2.5 × 10^4^ ± 1.2 × 10^4^ M^−1^ with an apparent stoichiometry (number of ligand binding sites) N of 1.4 ± 0.8. This corresponds to a dissociation constant *K*_d_ of around 40 μM, which is in reasonable agreement with the *K*_d_ of 8.25 μM obtained by NMR chemical shift perturbation. Due to the relatively large standard deviation, the values derived from this ITC experiment should be interpreted with caution. Qualitatively, however, the ITC experiment confirmed the conclusions of the NMR titration.

### 3.4 Analysis of anti-r CHIPS_28–149_ antibodies in patient and control sera

**Fig. 6:**
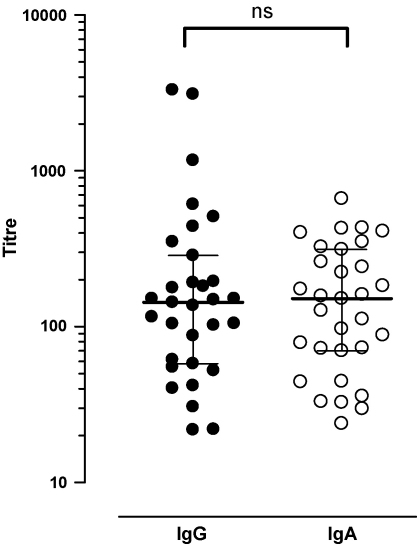
IgG and IgA have similar titres against CHIPS. IgG and IgA titres against recombinant CHIPS in control and patient sera were measured by ELISA and are shown as the dilution required to obtain half-maximal antibody binding measured in duplicate. The medians of the non-normally distributed titres are shown, with interquartile range. The difference between IgG and IgA titres was non-significant (*p* = 0.91) by the Mann–Whitney test.

**Fig. 7:**
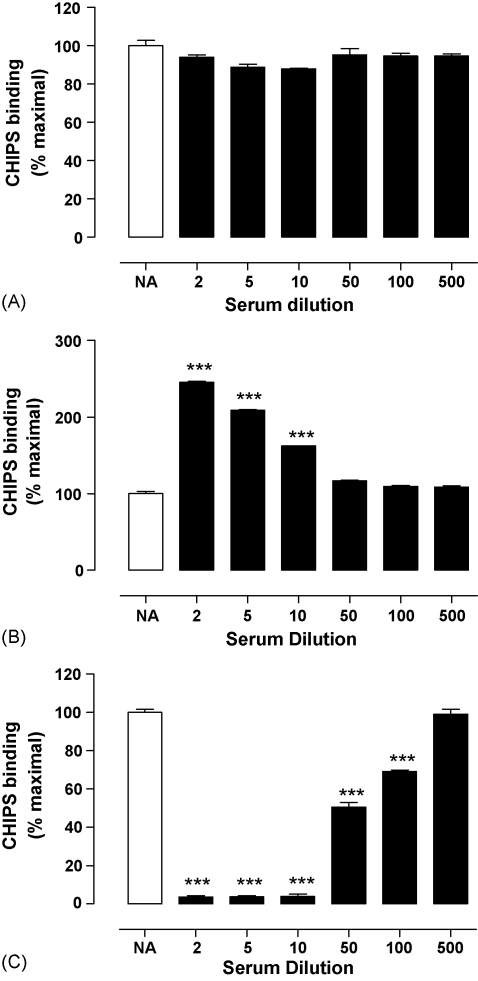
Effects of human sera on CHIPS binding. Examples of the effects of human sera on FITC-CHIPS binding to human C5a receptor: (A) serum 8 (no effect), (B) 20 (enhancement of binding), and (C) (inhibition of binding). Diluted sera were co-incubated with 210 nM FITC-labelled rCHIPS_28–149_, and binding measured by flow cytometry. NA represents FITC-rCHIPS_28–149_ binding in the absence of human serum. Data are the means ± S.D. from two experiments performed in triplicate, expressed as a percentage of rCHIPS_28–149_ binding in the absence of sera. Significance was assessed by *t* test; ^***^p < 0.0001.

**Fig. 8:**
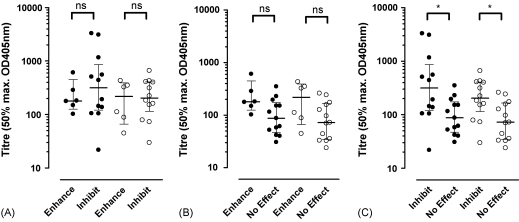
Relationship between anti-CHIPS titre and antibody effects on CHIPS binding to C5a receptor. The medians and interquartile ranges of the anti-CHIPS titres of inhibitory or enhancing IgG (filled circles) and IgA (open circles) content of control and patient sera was compared using Mann–Whitney tests comparing serum IgG and IgA titre to rCHIPS_28–149_ in ELISA, in relation to the effects of serum on FITC-labelled rCHIPS_28–149_ binding to RBL-2H3-C5aR cells. For this purpose, sera are grouped into those having inhibiting, enhancing, or no effect on rCHIPS_28–149_ binding.

**Fig. 9:**
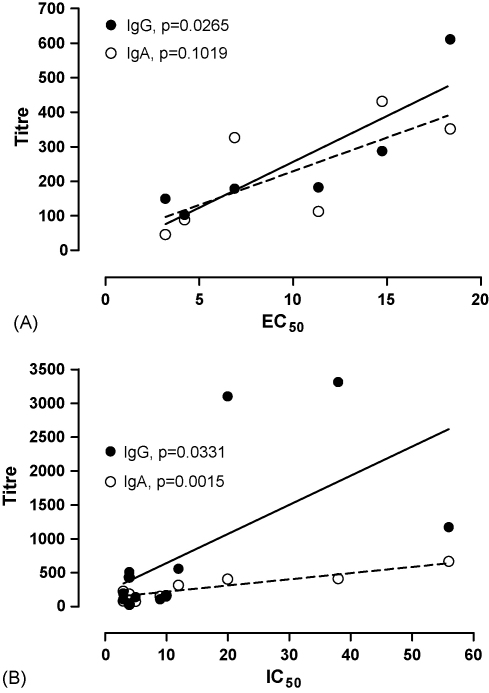
Correlation between anti-CHIPS titre and antibody effects on CHIPS binding to C5a receptor. The anti-CHIPS titres of IgG (filled circles, solid line) and IgA (open circles, broken line) plotted against half-maximal dilution factor for either the enhancement (A) or inhibition (B) of rCHIPS_28–149_ binding to C5a receptor. The significance of the difference of the slope value from 0 is shown.

As rCHIPS_28–149_ is clearly active at the C5aR, we were able to use this protein to analyse the levels and functional properties of the human IgG and IgA antibody response to CHIPS from 31 serum samples (7 *S. aureus* infected patients, 24 ‘normal’ donor individuals). Titres from each serum sample were determined by ELISA (Table 1). All serum samples contained rCHIPS_28–149_-reactive IgG and IgA but not IgM antibodies and the titres had approximately Gaussian distributions (Fig. 6). No difference could be observed in the overall IgG or IgA titre to rCHIPS_28–149_ (Fig. 6), or in the IgG or IgA response to CHIPS between infected (seven samples) or ‘normal’ donor groups (24 samples) (data not shown). To analyse the functional properties of these antibodies, serum samples were pre-incubated with FITC-labelled rCHIPS_28–149_ (210 nM—determined to give 50% maximal binding to C5aR in this instance) and the complexes assayed for binding to RBL-C5aR using flow cytometry. Some samples were also tested for binding to CHO-C5aR cells (since these lack Fc receptors which may potentially cause problems when using RBL-2H3 cells). However, values obtained for each cell line revealed comparable effects on rCHIPS_28–149_ binding, hence all subsequent assays involved the RBL-2H3-C5aR cell line. Three distinct groups of serum samples were identified (Table 1): (1) those which inhibit rCHIPS_28–149_ binding (12/32, 38%), (2) those which enhance rCHIPS_28–149_ binding (7/32, 22%) and (3) those which have no effect on rCHIPS_28–149_ binding (13/32, 40%) (Table 1); examples of each group are shown in Fig. 7. To further analyse these effects, IgG from sample 21 was purified using protein G and concentrated to ∼12 mg ml^−1^ (i.e. to approximately serum levels). This purified IgG clearly showed an inhibitory effect on rCHIPS_28–149_ binding similar to that displayed using 21 serum. This was also observed using purified 29 IgG (data not shown). To confirm the role of IgG antibodies on rCHIPS_28–149_ binding, sera from 7 samples subjects were depleted of IgG using protein G-linked sepharose. The IgG-depleted sera all showed reduced inhibitory/enhancing effects on rCHIPS_28–149_ binding (Table 2). The biggest difference observed was in the two serum samples which enhanced rCHIPS_28–149_ binding, 17 and 18; IgG depletion completely blocked the enhancing effect of 18 serum and significantly reduced 17 mediated enhancement. Residual activity observed following IgG depletion may be due to the presence of remaining IgA antibodies not removed by protein G treatment. To determine if the different functional properties of sera were due simply to the titre of IgG or IgA anti-rCHIPS_28–149_ antibodies, we compared titres of serum groups having enhancing, inhibiting, or no effect on rCHIPS_28–149_ binding (Fig. 8). No significant differences were observed for either IgG or IgA titres when comparing sera with enhancement versus inhibition groups or enhancement versus no effect groups on rCHIPS_28–149_ binding to RBL-C5aR cells, but both IgG and IgA titres were significantly different when comparing inhibition versus no effect groups. The role of titre in determining functional activity was further assessed by correlating titre within enhancement or inhibition groups (Fig. 9). The data from three of the four plots with the exception of IgA titre in the enhancement group binding show a positive correlation between antibody titre and ability to modify rCHIPS_28–149_ binding. Thus whilst the capacity to affect CHIPS activity relates to antibody titre, the inhibitory versus enhancing effects may relate to particular epitope specificities of antibodies within the sera.

## 4 Discussion

### 4.1 Structure-function studies on rCHIPS_28–149_

The data presented in this paper suggest that recombinant CHIPS_28–149_ is an effective antagonist of human C5aR. FITC-labelled protein was found to bind to human C5aR expressed on RBL-C5aR cells with an IC_50_ of ∼70 nM, which is similar to previously observed values (de Haas et al., 2004). The value we obtained for C5a-mediated inhibition of FITC-labelled CHIPS binding to the C5aR (∼250 nM) is also similar to that previously published (Postma et al., 2004), which reported 50% maximal inhibition at around ∼100 nM. The binding was relatively specific to C5aR, as no binding was detected to other chemoattractant receptors (ChemR23, FPRL1, FPRL2) and only low levels of binding to the recently identified second C5a receptor, C5L2. This is surprising because of the conservation of acidic and tyrosyl residues that make up the ligand binding site in the N-terminal domains of both receptors, including the residues 10–18 within the C5aR (Postma et al., 2005) proposed to form the binding site for CHIPS. C5L2 has been characterised as an anti-inflammatory decoy receptor that removes C5a and C5a des Arg from the circulation, thus preventing an excessive inflammatory response (Gao et al., 2005; Huber-Lang et al., 2005). It is likely that the C5aR binding site on CHIPS has evolved to become specific for the pro-inflammatory C5a receptor, C5aR, because activity at C5L2 would negate the antagonism at C5aR. However, rCHIPS_28–149_ clearly retains some activity at C5L2 and could inhibit the binding of C5a des Arg but not intact C5a to C5L2. C5L2 binds both of these ligands with nearly equal affinity and so the disproportionate effect on C5a des Arg binding may suggest that C5L2 binds C5a through a distinct binding site. However, the high concentration of rCHIPS_28–149_ required to inhibit C5a des Arg binding suggests that antagonism at C5L2 is unlikely to occur in vivo.

The lack of binding to, or antagonism of, FPR is almost certainly due to the three residues added to the N-terminus of rCHIPS_28–149_ following TEV protease cleavage of the GST-CHIPS_28–149_ fusion. We confirmed this by using rCHIPS_26–141_, which lacks this N-terminal extension, in a cell activation assay. Here, rCHIPS_26–141_ but not rCHIPS_28–149_ could inhibit the activation of FPR by formyl peptide. For the first time, we have shown that inhibition by CHIPS is reversible and competitive with an indication of negative cooperativity for both C5aR and FPR. The competitive nature of CHIPS antagonism of C5aR is not surprising because the CHIPS binding site at the N-terminus includes several of the residues associated with C5a binding (Chen et al., 1998; Mery and Boulay, 1993). Negative cooperativity may occur due to the reported dimerization of C5aR (Klco et al., 2003); if the N-termini of the dimerized C5aR are in close proximity, then binding of a second CHIPS molecule may be hindered. The kinetics of CHIPS binding to C5aR have also been analysed here for the first time, using a flow cytometric method. Binding is rapid and saturable within 2 min with a similarly fast dissociation rate; the *K*_d_ calculated from these rates is similar to that calculated from the competition binding studies reported here and elsewhere (Postma et al., 2004). These rapid kinetics support the notion of a reversible, competitive mode of antagonism.

The binding of rCHIPS_28–149_ to C5aR was further defined by structural studies. NMR chemical shift perturbation mapping, backed up by isothermal titration calorimetry, demonstrated that rCHIPS_28–149_ binds to a peptide corresponding to the extracellular N-terminal region of the human C5a receptor but does not bind to peptides corresponding to the second extracellular loop of hC5aR or the extracellular N-terminal region of the C5aR homologue C5L2. When mapped on to the structure of rCHIPS_31–121_ (Haas et al., 2005), the two regions of rCHIPS_28–149_ identified by NMR to be most important for interaction with the hC5aR_1–37_ peptide, residues 71–91 and 123–140, form a continuous region on the CHIPS surface (Fig. 5b). This binding region comprises a central negatively charged area surrounded by positive charges (Fig. 5b). Sequence comparison of human and mouse C5aR and human and mouse C5L2 shows that human C5aR has two unique basic residues, Lys 17 and Lys 28, within the N-terminal 30 residues. C5aR-C5a binding involves ionic interactions between negatively charged residues on C5aR and positive charges on C5a. In the C5aR-CHIPS interaction, it is possible that the negatively charged site at the centre of the CHIPS binding interface interacts with Lys 17 and/or Lys 28 of C5aR and that this favourable interaction helps to account for the greater inhibitory effect of CHIPS on human C5aR over mouse C5aR (Postma et al., 2004).

### 4.2 Human antibody response to CHIPS

A measurable IgG and IgA anti-CHIPS response was observed in all serum samples (24 ‘normal’ donor controls and 7 *S. aureus* infected samples) examined. To our knowledge this is the first report of antibodies to CHIPS in the general sera. Rooijakkers et al. (2005a,b) have recently reported the presence of IgG antibodies to Staphylokinase and a staphylococcal complement inhibitor over a wide concentration range in sera from a large number of donor and *S. aureus* infected samples. These antigens have also recently been identified and localised to the same *S. aureus* pathogenicity island as CHIPS, SaPI5, and like CHIPS are thought to play a role in staphylococcal virulence. Statistical analysis of the data shows no significant differences in the titres of anti-CHIPS antibodies between donor or infected individuals. Based on their effects on FITC-labelled rCHIPS_26–141_ binding to C5aR, sera were categorised into three groups, (1) those that enhance CHIPS binding (22%), (2) those that inhibit CHIPS binding (38%), and (3) those sera that have no effect on CHIPS binding (40%). There is clear evidence that the effects on CHIPS binding are antibody-mediated, since IgG purified from serum that inhibited CHIPS binding to C5aR was also inhibitory and IgG depletion substantially abrogated the ability of serum to enhance or inhibit CHIPS binding. This indicates that specific anti-CHIPS antibodies might affect CHIPS activity in vivo. There was a significant difference in IgG and IgA titres between groups of sera that inhibited CHIPS binding and those that had no effect. However, there was no significant difference in anti-CHIPS titres between the groups that enhanced or inhibited CHIPS binding. Futhermore, although there was a positive correlation between antibody titre and the capacity of the sera to affect CHIPS binding to C5aR, titre did not relate to whether the effects were enhancing or inhibitory. The difference between these two groups of sera therefore appears to relate to the fine specificities of the anti-CHIPS antibodies they contain. Interestingly, 72% of sera from infected individuals inhibit rCHIPS_26–141_ binding to the C5aR, whereas only 28% of sera from ‘normal’ donors are inhibitory. Since there are only seven samples in the *S. aureus* infected group, further testing is clearly required to determine the immunological significance of this finding. However, we speculate that the ability to block CHIPS binding to C5aR may provide the host with a significant advantage as this would facilitate binding of C5a, thus stimulating leukocyte recruitment and chemotaxis towards the site of *S. aureus* infiltration. Antibodies that enhance CHIPS binding have a more ambiguous role in disease aetiology. It is conceivable that some anti-CHIPS antibodies bind the C5aR binding domain of CHIPS thus blocking CHIPS binding, while others bind CHIPS at another site that facilitates binding to C5aR, perhaps through multimerization. The finding by Haas et al. (2005) that immunisation of mice with recombinant CHIPS produces antibodies with a range of binding specificities for the CHIPS molecule may substantiate this theory. It would be of considerable interest to identify the epitope(s) recognised by antibodies that inhibit CHIPS binding, since such antibodies might be developed as therapeutic reagents.

In conclusion, our studies have confirmed recent results demonstrating that CHIPS is able to bind C5aR with high affinity and specificity, and to inhibit the binding of C5a to this receptor by interacting with the receptor N-terminus. In addition, we have shown for the first time that CHIPS is highly immunogenic, with the identification of antibodies to CHIPS throughout the general population. Clearly, this questions the use of CHIPS as an anti-inflammatory therapeutic reagent as suggested by de Haas et al. (2004). In contrast, antibodies that inhibit CHIPS binding may have potential in treating or preventing *S. aureus* infection.

## Figures and Tables

**Table 1:** Analysis of anti-CHIPS IgG and IgA antibody titres of serum samples

Serum	Donor status	IgG titre	IgA titre	Effect on CHIPS binding	Change in CHIPS binding (%)^a^	EC_50_/IC_50_ (reciprocal dilution)
1	‘Normal’	52	24	No effect	N/A	N/A
2	‘Normal’	116	157	No effect	N/A	N/A
3	‘Normal’	229	127	No effect	N/A	N/A
4	‘Normal’	58	70	No effect	N/A	N/A
5	‘Normal’	351	261	No effect	N/A	N/A
6	‘Normal’	40	242	No effect	N/A	N/A
7	‘Normal’	42	33	No effect	N/A	N/A
8	‘Normal’	62	44	No effect	N/A	N/A
9	‘Normal’	31	33	No effect	N/A	N/A
10	‘Normal’	195	174	No effect	N/A	N/A
11	‘Normal’	151	73	No effect	N/A	N/A
12	‘Normal’	151	97	No effect	N/A	N/A
13	‘Infected’	87	36	No effect	N/A	N/A
14	‘Normal’	178	326	Enhancing	208	7
15	‘Normal’	182	112	Enhancing	170	11
16	‘Normal’	149	45	Enhancing	135	3
17	‘Normal’	610	351	Enhancing	386*	118
18	‘Normal’	287	431	Enhancing	214*	15
19	‘Normal’	ND	ND	Enhancing	245	9
2	Infected	102	88	Enhancing	155	4
21	‘Normal’	555	313	Inhibiting	13	12
22	‘Normal’	22	30	Inhibiting	66	4
23	‘Normal’	440	184	Inhibiting	7	4
24	‘Normal’	143	161	Inhibiting	32	10
25	‘Normal’	508	428	Inhibiting	17	4
26	‘Normal’	192	224	Inhibiting	26	3
27	‘Normal’	3097	402	Inhibiting	4	20
28	Infected	105	79	Inhibiting	27	3
29	Infected	3308	411	Inhibiting	3	38
30	Infected	105	152	Inhibiting	49	9
31	Infected	137	73	Inhibiting	12	5
32	Infected	1168	663	Inhibiting	4	56

a Change in CHIPS binding relative to control (=100%) at 1/2 dilution of serum except for * at 1/5 dilution.

**Table 2:** Effects of IgG depletion on the ability of sera to modulate CHIPS binding to the human C5a receptor

Serum (effect on CHIPS binding)	Change in CHIPS binding (%)^a^	
	Untreated Serum	IgG-depleted serum
21 (inhibiting)	13	41
29 (inhibiting)	3	67
30 (inhibiting)	49	71
31 (inhibiting)	12	83
17 (enhancing)	386	121
18 (enhancing)	214	100
27 (inhibiting)	4	76

a Change in CHIPS binding relative to control (=100%) at 1/2 dilution of serum.
